# Sepsis-Induced Cardiac Mitochondrial Dysfunction Involves Altered Mitochondrial-Localization of Tyrosine Kinase Src and Tyrosine Phosphatase SHP2

**DOI:** 10.1371/journal.pone.0043424

**Published:** 2012-08-27

**Authors:** Qun S. Zang, Bobbie Martinez, Xiao Yao, David L. Maass, Lisha Ma, Steven E. Wolf, Joseph P. Minei

**Affiliations:** Departments of Surgery, University of Texas Southwestern Medical Center, Dallas, Texas, United States of America; University of Western Ontario, Canada

## Abstract

Our previous research demonstrated that sepsis produces mitochondrial dysfunction with increased mitochondrial oxidative stress in the heart. The present study investigated the role of mitochondria-localized signaling molecules, tyrosine kinase Src and tyrosine phosphatase SHP2, in sepsis-induced cardiac mitochondrial dysfunction using a rat pneumonia-related sepsis model. SD rats were given an intratracheal injection of *Streptococcus pneumoniae*, 4×10^6^ CFU per rat, (or vehicle for shams); heart tissues were then harvested and subcellular fractions were prepared. By Western blot, we detected a gradual and significant decrease in Src and an increase in SHP2 in cardiac mitochondria within 24 hours post-inoculation. Furthermore, at 24 hours post-inoculation, sepsis caused a near 70% reduction in tyrosine phosphorylation of all cardiac mitochondrial proteins. Decreased tyrosine phosphorylation of certain mitochondrial structural proteins (porin, cyclophilin D and cytochrome C) and functional proteins (complex II subunit 30kD and complex I subunit NDUFB8) were evident in the hearts of septic rats. *In vitro*, pre-treatment of mitochondrial fractions with recombinant active Src kinase elevated OXPHOS complex I and II-III activity, whereas the effect of SHP2 phosphatase was opposite. Neither Src nor SHP2 affected complex IV and V activity under the same conditions. By immunoprecipitation, we showed that Src and SHP2 consistently interacted with complex I and III in the heart, suggesting that complex I and III contain putative substrates of Src and SHP2. In addition, *in vitro* treatment of mitochondrial fractions with active Src suppressed sepsis-associated mtROS production and protected aconitase activity, an indirect marker of mitochondrial oxidative stress. On the contrary, active SHP2 phosphatase overproduced mtROS and deactivated aconitase under the same *in vitro* conditions. In conclusion, our data suggest that changes in mitochondria-localized signaling molecules Src and SHP2 constitute a potential signaling pathway to affect mitochondrial dysfunction in the heart during sepsis.

## Introduction

Severe sepsis, associated with staggering inflammatory responses and multi-organ failure, is a leading cause of death in intensive care units [1], [2]. Despite improvements in antibiotic therapies and critical care techniques [3], there are still approximately 215,000 Americans die from sepsis each year [4]. The understanding of sepsis pathophysiology and our therapeutic options are still limited.

Among many intracellular players that contribute to the pathogenesis of sepsis, mitochondrial functional deficiency and mitochondrial reactive oxygen species (mtROS) overproduction are commonly recognized as major promoters [5], [6]. In patients with severe sepsis, the degree of mitochondrial dysfunction in skeletal muscle and liver biopsies has been found to associate with clinical outcomes [7], [8]. The underlying mechanism of mitochondrial function in sepsis pathogenesis probably involves multiple pathways. Impaired mitochondria respiration has been proposed to cause tissue level defect of oxygen utilization, termed “cytopathic hypoxia”, during sepsis-mediated organ failure [9], [10]. Imbalanced mtROS production due to altered mitochondrial metabolism directly cause mitochondrial structural and functional damage [11], [12] and also contribute to overall intracellular oxidative stress to produce cellular injuries [13]–[15]. Furthermore, recent discoveries implicated mitochondria in sepsis-induced inflammation. Innate immunity utilizes mtROS as a trigger to activate inflammasome NLRP3 in macrophages [16]. Mitochondrial matrix protein MAVS is part of the mitoxosome to activate NF-κB during antiviral responses [17]. In the plasma from trauma patients, circulating mtDNA fragments released from damaged mitochondria were identified as mitochondria-derived danger-associated molecular patterns (DAMPs) to trigger peripheral inflammation [18]. To date, intracellular molecular pathways that lead to mitochondrial dysfunction during sepsis have not been understood, and research in this area is expected to reveal the mechanism of the disease and to identify potential therapeutic targets.

A growing body of evidence suggests that reversible phosphorylation of mitochondrial proteins plays an essential part in control of mitochondrial function and structure [19]–[22]. Proteomic analysis captured phosphorylation sites on critical enzymes of mitochondria metabolism, membrane components and biosynthesis molecules in healthy mitochondria isolated from rat brains [23] and from mouse hearts [24]. Recent investigations implicated certain well-known intracellular signaling molecules, such Src-family tyrosine kinases [25], tyrosine phosphatases PTP-1B and SHP2 [20], and serine/threonine kinases, protein kinase C (PKC) [26], [27] and extracellular-signal-regulated kinases (ERK) [22], [28], in the regulation of protein phosphorylation and dephosphorylation inside mitochondria. These molecules do not possess mitochondria-sorting peptide and the mechanism of their mitochondria translocation is not understood yet. However, their intra-mitochondria localization was verified using immune electron microscopy [25], [28], [29] and western blot analysis [20], [25]. Currently, the functional significance of mitochondria-localized kinases and phosphotases in sepsis-mediated mitochondrial damage in different organs is not known.

Cardiac dysfunction is an important component of multi-organ failure induced by severe sepsis [30]–[32]. Septic patients with cardiac dysfunction have significantly higher mortality compared with patients without this condition (70 *vs.* 20%) [33], [34]. In the heart, mitochondria comprise about 30% of myocardial volume [35]. Mitochondrial dysfunction, such as impaired metabolism, altered energy generation and elevated production of ROS, has been implicated in promoting sepsis-associated myocardial injury [36]–[38]. Previously, our laboratory developed a pneumonia-related sepsis model in rats [39]. In this model, rats were infected with *S. pneumoniae* and sepsis symptoms were confirmed by positive blood cultures, pulmonary inflammation, lactic acidosis, and a fall in mean arterial blood pressure 24 hours post-infection [40]–[43]. Using this model, we demonstrated that sepsis impaired cardiac mitochondria, causing compromised membrane integrity, increased oxidative stress, and decreased antioxidant defense [44]. Our recent application of a mitochondria-targeted antioxidant provided direct evidence to support that mtROS-mediated mitochondria impairment plays a causative role in myocardial inflammation and cardiac dysfunction during sepsis [45]. In this report, we investigated the function of intracellular signaling molecules, tyrosine kinase Src and tyrosine phosphatase SHP2, in mitochondrial impairment in the heart using the rat pneumonia-related sepsis model.

## Results

### Sepsis Alters Mitochondrial Translocation of Tyrosine Kinase Src and Phosphatase SHP2 in the Heart

**Figure 1:**
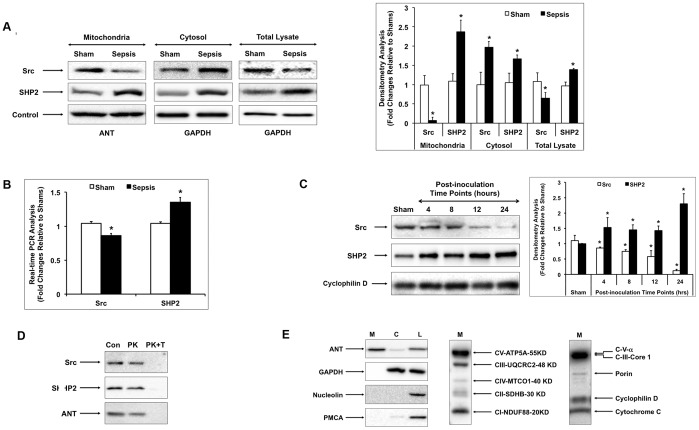
Sepsis alters subcellular distribution and total expression levels of tyrosine kinase Src and tyrosine phosphatase SHP2 in the heart. Sprague-Dawley rats were infected by *S. pneumoniae* or given PBS sham control. Rats were killed at indicated time points and subcellular fractions were prepared from the heart tissues. **A.** Changes in subcellular distribution of Src and SHP2 in myocardium 24 hours post-inoculation. Protein samples of mitochondria, cytosol and total heart tissue lysates were analyzed by Western blot using antibodies against Src, SHP2, mitochondrial marker protein adenine nucleotide translocase (ANT), and cytosolic marker glyceraldehyde 3-phosphate dehydrogenase (GAPDH). Results were quantified by densitometry analysis and expressed as fold changes relative to shams. **B.** Changes in total mRNA levels of Src and SHP2 in myocardium 24 hours post inoculation. Real-time PCR using commercially available primer-probe sets for Src and SHP2 was performed in triplicate in all samples, and results were normalized to the housekeeping gene GAPDH. **C.** Time course changes of mitochondria-localized Src and SHP2 in the heart after sepsis. Protein samples of cardiac mitochondria from sham and sepsis rats killed at indicated post-inoculation time points were analyzed by Western blot using antibodies against Src, SHP2 and mitochondrial matrix protein cyclophilin D. Results were quantified by densitometry analysis and expressed as fold changes relative to shams. In A-C, all values are means ±SE. Statistical significances between sham and sepsis subjects are labeled with * (*p*<0.05, n = 6 per group). **D.** Confirmation of Src and SHP2 intra-mitochondria localization by proteinase K sensitivity. Freshly isolated mitochondria fractions were treated with 100 ng/ml proteinase K (PK) in the absence or presence of 0.5% Triton X-100 (T) at room temperature for 30 min followed by Western blot analysis using antibodies against Src, SHP2 or ANT. **E.** Confirmation of mitochondria isolation. Mitochondrial (M), cytosolic (C) fractions or total heart tissue lysates (L) were analyzed by Western blot using antibodies against marker proteins ANT, GAPDH, plasma membrane-type Ca^2+^-ATPase (PMCA), and nucleolin. Mitochondrial respiratory complexes (I-V) and mitochondrial membrane integrity markers were examined in the mitochondrial fractions (M) using cocktail antibodies. Data shown in both **D** and **E** are representative of at least 4–6 animals per group.

To determine whether sepsis changes the expression and subcellular distribution of tyrosine kinase Src and phosphatase SHP2 in the heart, we examined their levels in mitochondria, cytosol and total tissue lysates by Western blot in the heart tissue harvested 24 hours post bacterial inoculation. As shown in Figure 1A, sepsis caused a dramatic ∼80% reduction of Src and a more than one fold increase of SHP2 in mitochondria. As a control, mitochondrial marker adenine nucleotide translocase (ANT) showed no difference between sham and sepsis samples. In parallel, sepsis increased both Src and SHP2 levels in the cytosol while had no effect on cytosolic marker glyceraldehyde-3-phosphate dehydrogenase (GAPDH). When whole tissue lysates were examined, a significant ∼35% decrease in Src and a ∼40% increase in SHP2 expression were found to be associated with sepsis. An additional real-time PCR analysis revealed similar differences of Src and SHP2 mRNA expression between sham and sepsis rats, indicating that sepsis-mediated changes of Src and SHP2 expression in the heart were regulated at the transcription level (Figure 1B).

The sepsis-induced variations in mitochondria-localized Src and SHP2 were further analyzed in a time course study (Figure 1C). In the mitochondrial fractions isolated from the hearts harvested at 4, 8, 12 and 24 hours post-inoculation, a significant decrease in Src and an increase in SHP2 were detected as early as 4 hours following the septic challenge. These changes were further progressed and reached to the maximal levels at 24 hours post inoculation. As controls, no significant variation was shown for mitochondrial matrix protein cyclophilin D and cytosolic GAPDH was not detectable in these mitochondrial protein samples (data not shown).

The intra-mitochondria localization of Src and SHP2 was further verified by the insensitivity of these molecules to proteinase K treatment. As shown in Figure 1D, isolated heart mitochondria were treated with proteinase K in the presence or absence of detergent Triton X-100 (TX-100) and subsequently analyzed by Western blot. In all these samples, presence of cytosol marker GAPDH was negative (data not shown), confirming the complete separation of mitochondria from cytosol. Similar to mitochondrial marker ANT that resides in the inner mitochondrial membrane [46], [47], majority of Src and SHP2 was resistant to proteinase K digestion. Our previous publications revealed that basal level membrane damage of mitochondria isolated from healthy heart tissue was about 10%–15%, presumably caused by the isolation procedure itself [44], [45], [48], [49]. Therefore, since this amount of non-intact mitochondria was digested by proteinase K, slight decrease of Src, SHP2 and ANT after proteinase K treatment was expected. In the presence of detergent TX-100, Src, SHP2 and ANT were completely degraded and no longer detectable. Taken all, the result presented here indicates that Src and SHP2 are present inside mitochondria in the heart tissue.

In these experiments, the quality of mitochondrial preparations was verified by Western blot using antibodies against multiple markers of subcellular fractions. As shown in Figure 1E, mitochondrial marker ANT and cytosol marker GAPDH were exclusively present in the mitochondrial and cytosol fractions respectively. Consistently, cell membrane protein, plasma membrane-type Ca^2+^-ATPase (PMCA), or nuclear protein, nucleolin, was merely detectable in these fractions. All these markers appeared in the total tissue lysates. In addition, the presence of mitochondrial respiratory complexes (I-V) and markers of mitochondrial membrane integrity was confirmed in the mitochondrial fractions.

### Sepsis Reduces Tyrosine Phosphorylation of Mitochondrial Functional and Structural Proteins in the Heart

**Figure 2:**
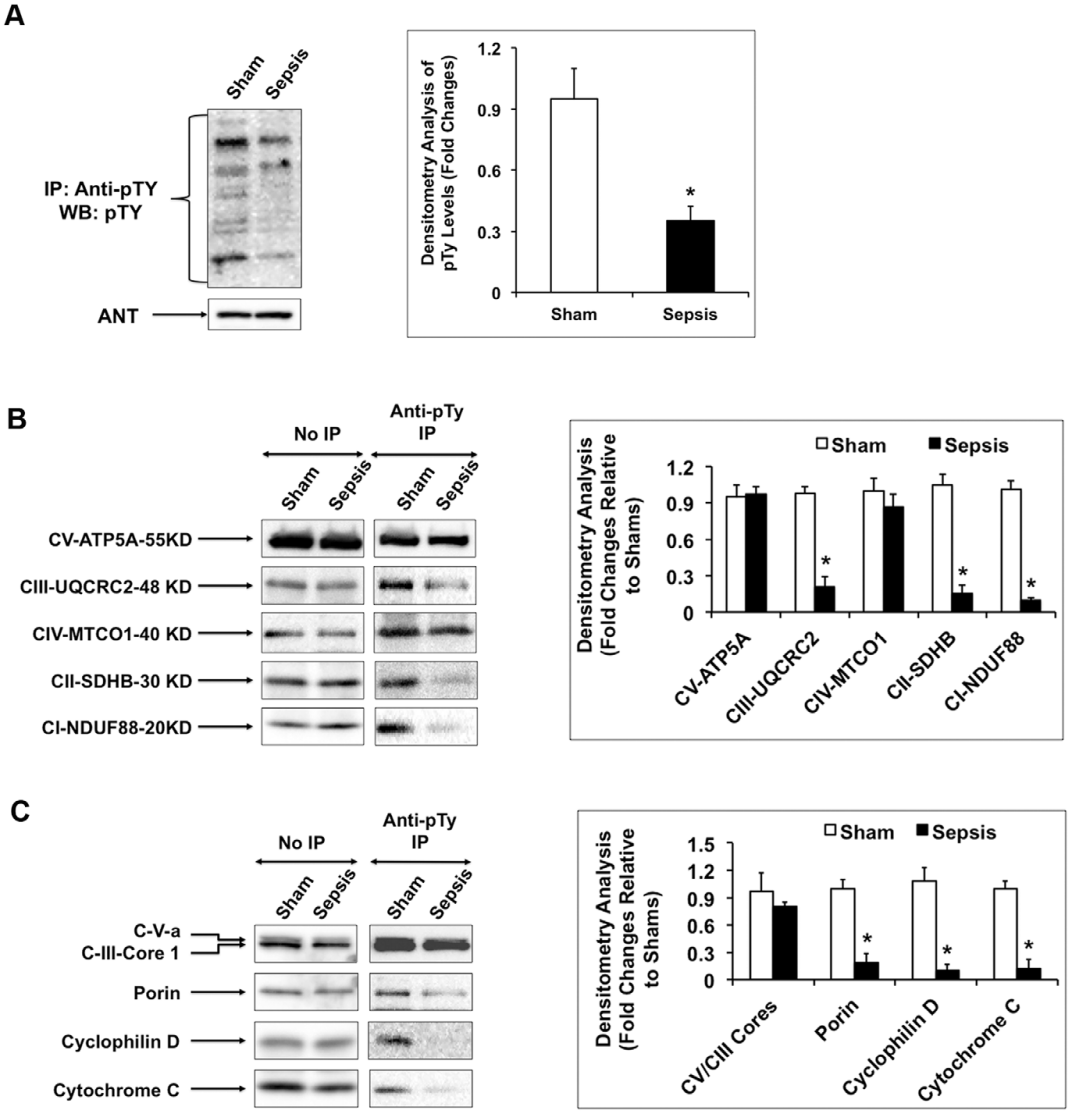
Sepsis decreases tyrosine phosphorylation of mitochondrial proteins in the heart after sepsis. Rats were infected by *S. pneumoniae* or given PBS sham control, killed 24 hours post-inoculation and mitochondrial fractions were isolated from the heart tissues. Pooled mitochondria preparations (n = 3 rats) were solubilized by 1 mM n-dodecyl-ß-D-maltopyranoside, subjected to immunoprecipitation with anti-phosphotyrosine-sepharose 4B, and analyzed by Western blot using anti-phosphotyrosine (**A**), antibody cocktails against mitochondrial OXPHOS complexes (**B**), or antibody cocktails against mitochondrial membrane proteins (**C**). Data shown are representative of at least 3 independent experiments. Results were quantified by densitometry analysis and expressed as fold changes relative to shams. All values are means ±SE. Statistical significances between sham and sepsis subjects are labeled with * (*p*<0.05, n = 6 per group).

We next examined whether sepsis alters tyrosine phosphorylation of mitochondrial proteins in the heart. Mitochondrial fractions were isolated from the hearts of sham and sepsis rats 24 hours post-inoculation. Tyrosine phosphorylated proteins were extracted by immunoprecipitation and analyzed by Western blot using anti-phosphotyrosine (Figure 2A). Compared with sham controls, sepsis rats showed a near 70% decrease of tyrosine phosphorylation level in total cardiac mitochondrial proteins. Furthermore, using an antibody cocktail against marker proteins of mitochondrial oxidative phosphorylation (OXPHOS) complex I-V (Fig. 2B), we found that sepsis reduced ∼80% tyrosine phosphorylation of NDUF88 in complex I, succinate dehydrogenase complex, subunit B (SDHB) in complex II, and ubiquinol-cytochrome c reductase core protein II (UQCRC2) in complex III. Phosphorylation of mitochondria-encoded cytochrome c oxidase I (MTCO1, also known as COX1) in complex IV and ATP5A in complex V (ATP synthase) was not affected by sepsis. Using an antibody cocktail against marker proteins of mitochondrial membrane (Fig. 2C), we detected a similar degree of reduction in tyrosine phosphorylation of porin, cyclophilin D and cytochrome C. On the contrary, tyrosine phosphorylation of complex V (C-V) alpha subunit and complex III (C-III) core 1 did not respond to sepsis. Taken all together, our study showed that, in the heart, sepsis significantly decreases tyrosine phosphorylation of mitochondrial functional and structural proteins.

### Sepsis-mediated OXPHOS Deficiency in Cardiac Mitochondria Involves Src and SHP2

**Figure 3:**
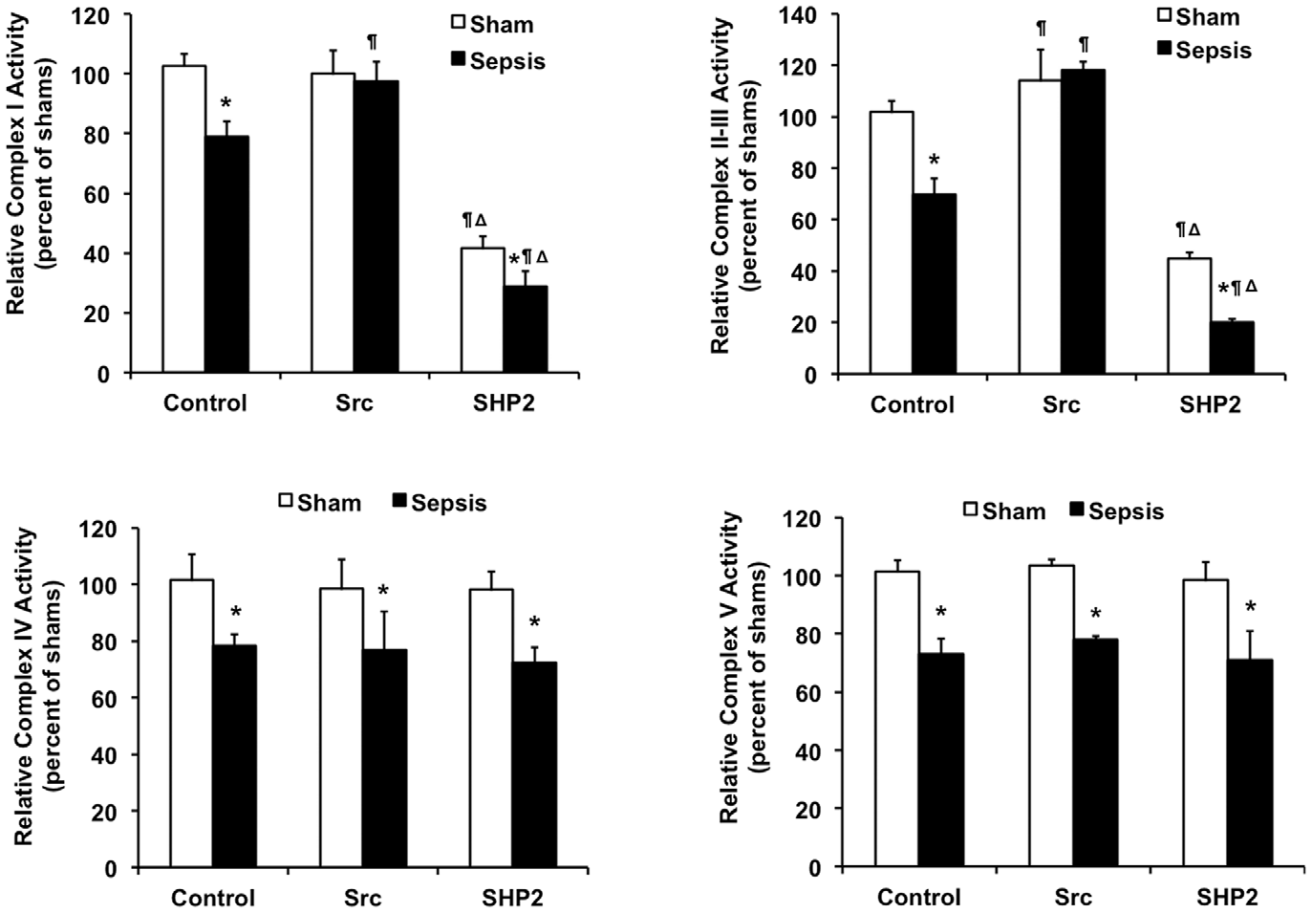
Cardiac mitochondrial OXPHOS activities regulated by active Src and SHP2 *in vitro*. Rats were infected by *S. pneumoniae* or given PBS sham control and killed 24 hours post-inoculation. Mitochondrial fractions were isolated from the heart tissues, treated with active Src or SHP2, and subjected to the measurements of OXPHOS complex I-V activities. Mitochondria without treatment were used as control. Results are expressed as percentage of shams. All values are means ±SE. Statistical significances: * difference between sham and sepsis, ¶ difference between control and treatments, Δ difference between Src and SHP2 (*p*<0.05, n = 6 per group).

To determine if mitochondria-localized Src kinase and SHP2 phosphatase have any function in the regulation of mitochondrial metabolism in the heart during sepsis, we examined whether recombinant active enzyme of Src or SHP2 affected the activities of mitochondrial OXPHOS complexes I-V *in vitro*. As shown in Figure 3, enzymatic activities of complex I, II-III, IV and V were measured in mitochondrial fractions isolated from the heart tissue of sham and sepsis rats 24 hours post-inoculation. Sepsis caused an overall ∼20–30% decrease of these OXPHOS activities. Pre-treatment of mitochondrial proteins with active Src inhibited sepsis-mediated reduction of complex I activity and significantly elevated complex II-III activity in both sham and sepsis subjects. On the contrary, treatment of mitochondria with active SHP2 dramatically led to a more than 50% reduction of these activities in all samples. Under the same condition, Src and SHP2 provided no effect on the activities of complex IV and V in mitochondrial preparations from either sham or sepsis rats.

**Figure 4:**
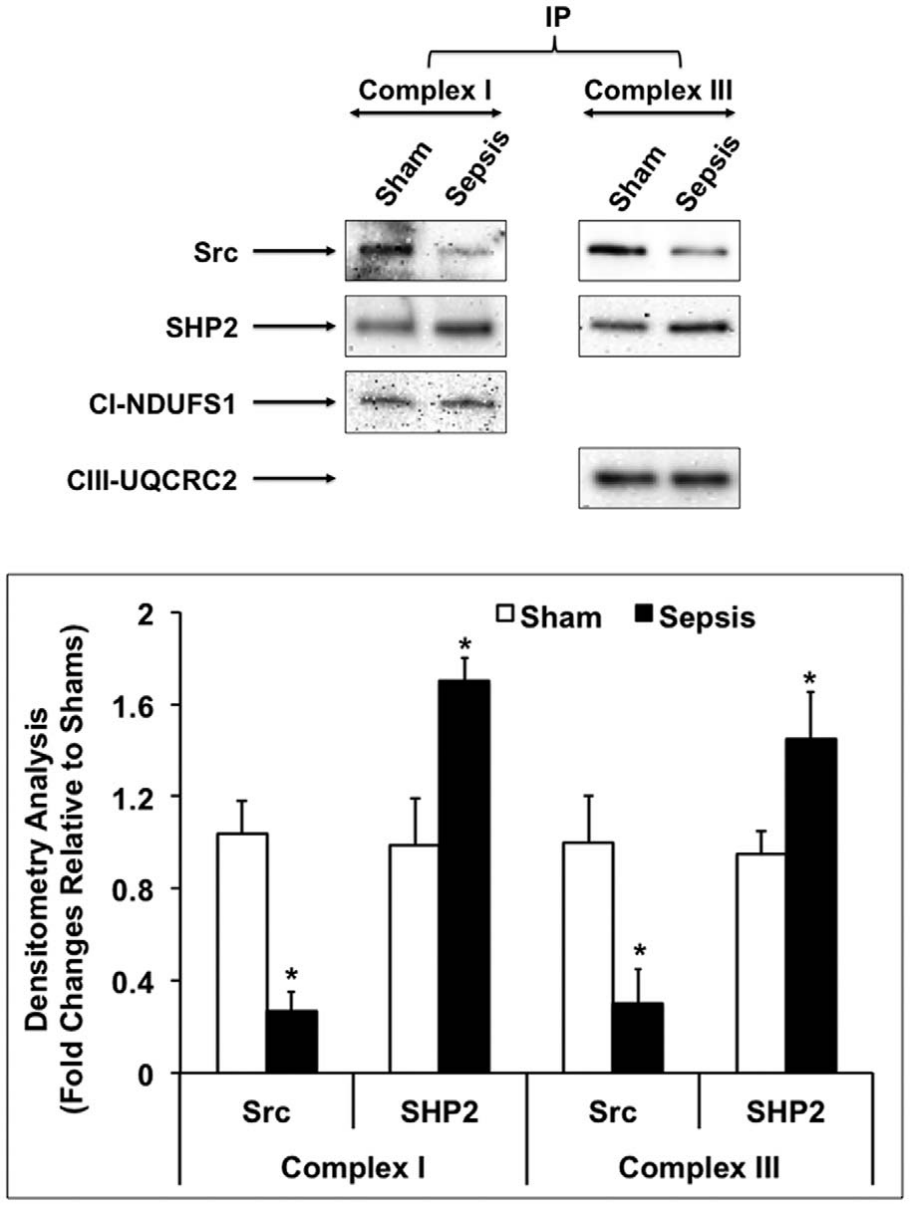
Association of Src and Shp2 with OXPHOS complexes I and III in cardiac mitochondria. Rats were infected by *S. pneumoniae* or given PBS sham control, killed 24 hours post-inoculation and mitochondrial fractions were isolated from the heart tissues. Pooled mitochondria preparations (n = 3 rats) were immunoprecipitated with immunocapture antibodies against complex I or complex III, and analyzed by Western blot using antibodies against Src, SHP2, complex I marker NDUFS1 and complex III marker UQCRC2. Data shown are representative of at least 3 independent experiments. Results were quantified by densitometry analysis and expressed as fold changes relative to shams. All values are means ±SE. Statistical significances between sham and sepsis subjects are labeled with * (*p*<0.05, n = 6 per group).

To explore the possibility that components of complex I, II or III are targets of Src and SHP2 in mitochondria, we examined whether Src and/or SHP2 were physically associated with these OXPHOS complexes *in vivo*. Mitochondrial fractions from the heart tissue of sham and sepsis rats were subjected to immunoprecipitation using antibodies against complex I-V, and the presence of Src and SHP2 in the pull-down proteins was examined by Western blot. As shown in Figure 4, Src and SHP2 interacted with complex I and III in mitochondria from both sham and sepsis animals. Sepsis caused a decrease of Src and an increase of SHP2 that associated with these OXPHOS complexes, which differences correlate with sepsis-induced changes of mitochondria-located Src and SHP2 (shown in Figure 1A). Thus, the interaction between Src/SHP2 and complex I/III appears to be persistent. No positive Src or SHP2 signal was detected when mitochondrial proteins were immunoprecipitated with antibodies against complex II, IV or V (data not shown). Our results suggest that complex I and III contain putative substrates of Src and SHP2.

### Effects of Active Src and SHP2 on Cardiac Mitochondrial Oxidative Stress *in vitro*

**Figure 5:**
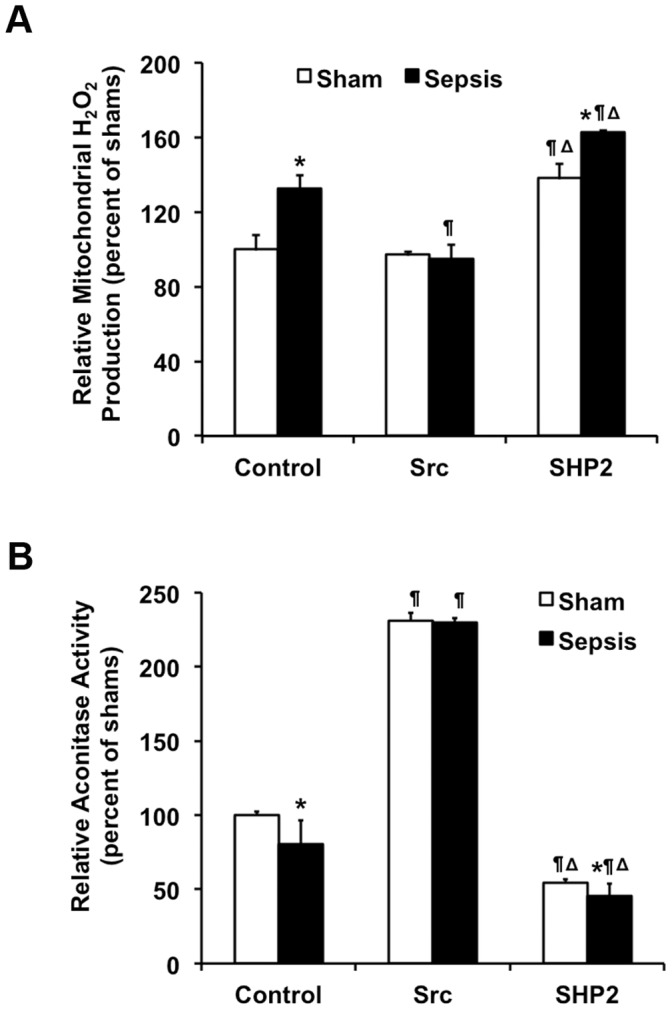
Regulation of mitochondrial oxidative stress by active Src and Shp2 *in vitro*. Rats were infected by *S. pneumoniae* or given PBS sham control and killed 24 hours post-inoculation. Mitochondrial fractions were isolated from the heart tissues, treated with active Src or SHP2. Mitochondria without treatment were used as control. **A.** Mitochondrial generation of H_2_O_2_ was measured by an Amplex Red fluorescence assay. The amount of H_2_O_2_ per reaction was calculated according to a standard curve and results are presented as percentage of shams. **B.** Aconitase activities were measured and normalized by the amount of mitochondrial proteins per reaction. Results are expressed as percentage of shams. All values are means ±SE. Statistical significances: * difference between sham and sepsis, ¶ difference between control and treatments, Δ difference between Src and SHP2 (*p*<0.05, n = 6 per group).

To evaluate the possibility that mitochondrial Src kinase and SHP2 phosphatase regulate mitochondrial ROS (mtROS) signaling in the heart during sepsis, we examined the effects of *in vitro* active Src and SHP2 on mtROS productivity and mitochondrial aconitase activity. To compare mtROS, mitochondrial fractions from sham and sepsis rats were supplemented with superoxide dismutase to convert superoxide to H_2_O_2,_ and subsequently subjected to a standard Amplex Red assay to quantify total H_2_O_2_ levels [45], [50]. As shown in Figure 5A, sepsis triggered H_2_O_2_ overproduction in mitochondria; a ∼30% increase in the presence of respiration substrate succinate (Figure 5A). However, this increase was abolished when mitochondria were pre-treated with active Src kinase *in vitro*. On the contrary to Src kinase, SHP2 phosphatase treatment dramatically elevated H_2_O_2_ production in all mitochondria samples.

Because aconitase is sensitive to ROS oxidation, loss of mitochondrial aconitase activity has been interpreted as an indirect marker of mtROS elevation [48], [51]. We measured aconitase activities in the mitochondrial fractions in the presence or absence of active Src or SHP2 as an alternative approach to compare Src/SHP2 effects on mtROS production in the heart. As expected, sepsis reduced approximately 20% of aconitase activity in cardiac mitochondria (Fig. 5B). *In vitro* treatment of Src kinase resulted in a more than 2-fold increase of aconitase activity in mitochondria of all subjects, whereas the effect of SHP2 phosphatase was completely opposite. Taken all together, our data suggest that Src and SHP2 participate in the regulation of ROS production in cardiac mitochondria during sepsis.

## Discussion

In a rat pneumonia-related sepsis model, we investigated whether sepsis alters mitochondrial translocation of certain intracellular kinases and phosphatases in the myocardium and whether these signaling molecules participate in the development of sepsis-mediated mitochondrial dysfunction in the heart. We found that sepsis induces a substantial decrease in tyrosine kinase Src and an increase in tyrosine phosphatase SHP2 in cardiac mitochondria (Figure 1). Correspondingly, tyrosine phosphorylation of mitochondrial proteins, including components of mitochondrial OXPHOS complexes and elements of mitochondrial membrane structure, was significantly reduced by 70–80% (Figure 2). *In vitro* effects of active Src and SHP2 on mitochondrial OXPHOS complex I and III activities (Figure 3) and *in vivo* interaction between Src/SHP2 and complex I/III (Figure 4) suggest that certain subunits of these two OXPHOS complexes are putative substrates of Src and SHP2. In addition, measurements of mtROS and mitochondrial aconitase activity in the presence of Src or SHP2 *in vitro* (Figure 5) indicate that, in mitochondria, Src and SHP2 are involved in the regulation of mtROS production and mitochondrial oxidative stress. Together, these results provide evidence to support the novel hypothesis that sepsis-induced mitochondrial damage is mediated through alteration of mitochondria-localized Src kinase and SHP2 phosphatase in the heart.

Current publications suggest that tyrosine phosphorylation is an essential part of regulation of cardiac mitochondrial function [52], [53]. In sepsis, mitochondrial structural damage and functional deficiency have been widely observed in experimental models and in clinical settings [18], [45], [54], [55]. However, to date, sepsis-associated changes in tyrosine phosphorylation of mitochondrial proteins have not yet been well studied. In this report, we observed a significant decrease in tyrosine phosphorylation of mitochondrial proteins in the heart in response to sepsis (Figure 2). This change coincides with sepsis-mediated elevation of tyrosine phosphatase SHP2 and reduction of kinase Src in mitochondria (Figure 1), suggesting that Src and SHP2 might be the main enzymes regulating tyrosine phosphorylation in cardiac mitochondria during sepsis. It was previously reported that Src family kinases are critical in mitochondrial tyrosine phosphorylation in rat brain [25] and heart [19]. Functional significance of mitochondria-associated SHP2 was also suggested in rat brain [20] and in endothelial cells [56]. However, since the mitochondrial substrates of Src and SHP2 have not been well characterized, we certainly cannot exclude other tyrosine kinases and phosphotases in the regulation of tyrosine phosphorylation of mitochondrial proteins in the heart during sepsis.

Some key components of mitochondrial OXPHOS complexes have been identified as targets of tyrosine phosphorylation [23], [52] and potential substrates of Src kinase [57]. Our observation of active Src and SHP2 effects on OXPHOS activities *in vitro* and the interaction between Src/SHP2 and complex I/III *in vivo* suggest that some subunits in complex I and III are putative substrates of Src and SHP2 (Figure 3 and 4). Previous studies using small molecule inhibitors in mitochondrial preparations implicated similar function of Src and SHP2 [20], [57]. However, *in vitro* Src and SHP2 did not affect complex IV and V activities in our experiment. This result is in conflict with reported Src inhibitor effects on complex IV and V activities in rat brain tissue [20], [58]. The discrepancy might be caused by organ- or disease-specific responses. Future proteomic analysis to identify mitochondrial substrates of Src and SHP2 will allow us to understand how Src and SHP2 regulate mitochondrial OXPHOS reactions.

Since mtROS are mostly generated from the reactions of OXPHOS complex I and III [59], and these complexes may contain substrates of Src and SHP2, it is not surprising to detect that active Src and SHP2 affect mitochondrial H_2_O*_2_* production and aconitase activity (Figure 5). It was previously reported that aconitase can be phosphorylated by Src family kinase Fgr *in vitro* [60]. Src-mediated elevation of aconitase activity might be a result of combined effects of Src inhibition of mtROS and Src phosphorylation of aconitase itself. However, whether aconitase is a target substrate of Src and/or SHP2 *in vivo* remains to be determined.

**Figure 6:**
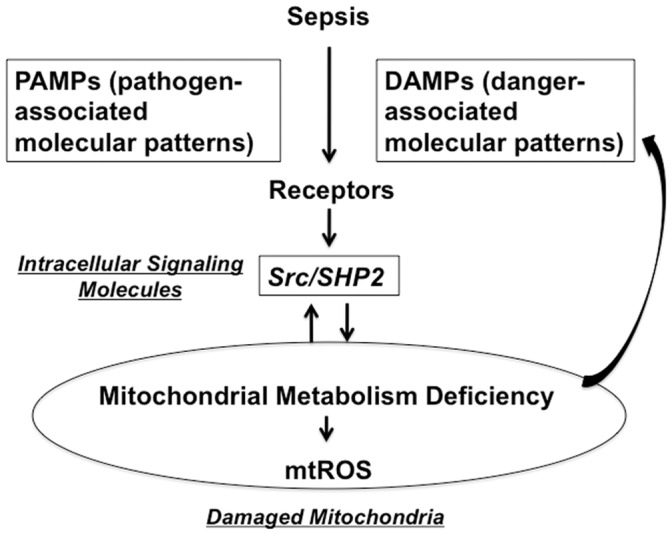
A proposed model of Src- and SHP2-mediated mitochondrial damage in the heart in response to sepsis.

We have previously shown that sepsis-induced mitochondrial oxidative stress is a causative factor for myocardial inflammation and cardiac dysfunction [44], [45]. Results presented in this report suggest that sepsis-mediated mitochondrial damage in the heart is regulated through a signal transduction pathway involving tyrosine kinase Src and phosphatase SHP2. As summarized in Figure 6, we hypothesize that, during sepsis, certain receptors of pathogen-associated molecular patterns (PAMPs) and/or danger-associated molecular patterns (DAMPs) alter mitochondrial translocation of Src and SHP2 in myocardium. The resulted changes in tyrosine phosphorylation of mitochondrial proteins produce functional deficiency and mtROS overproduction, and damaged mitochondria further generate more DAMPs to aggravate inflammatory responses and organ dysfunction [18]. In this model, several aspects need to be addressed in future studies. As mentioned above, mitochondrial substrates of Src and SHP2 remain to be defined. In addition, the upstream receptor(s) that regulates mitochondrial translocation of Src and SHP2 needs to be identified. Furthermore, whether alteration of mitochondrial Src and SHP2 relates to the production of mitochondrial-derived DAMPs to stimulate inflammation and how this signaling pathway affects cardiomyocyte function deserve further elucidation.

In this report, we also found sepsis-associated increase of Src kinase in myocardial cytoplasm (Figure 1A). Intracellular Src-mediated signal transduction pathway in the regulation of sepsis-induced cardiac dysfunction has not been fully understood yet. A recent proteomics analysis in H9C2 cardiomyocytes suggested that Src is a main kinase in the induction of tyrosine phosphorylation of intracellular proteins that are responsible for cell-cell connections, cell adhesion and morphology, leading to cardiac damage in response to oxidative injury [61]. In addition, previous studies using cardiomyocytes culture and heart tissue of different injury models implicated Src in the acute regulation of intracellular PH [62] and the induction of apoptosis [63], [64] in myocardium. Src was also linked to pathways of G-protein coupled receptors that regulate cardiac remodeling [65], [66]. In septic hearts, increased Src kinase in cytoplasm may exert similar roles in promoting heart failure. However, detailed analysis of sepsis-mediated changes in phosphorylation and activation status of Src and identification of potential targets of Src in the heart tissue are required in follow-up studies in order to further address the intracellular function of this kinase.

In summary, our current results of mitochondrial Src and SHP2 in the heart tissue of pneumonia-related sepsis rats revealed a plausible mechanism of how mitochondrial damage occurs in myocardium during sepsis. Further investigation of this mitochondrial Src/SHP2 pathway will promote the understanding of sepsis pathogenesis and will help to identify new therapeutic targets to control cardiac dysfunction.

## Materials and Methods

### Ethics Statement

Related animal protocol (protocol number 2011-0073) and pathogen safety plan were specifically approved by Institutional Animal Care and Use Committee (IACUC), ethics committee, and the department of Environmental Health and Safety (EH&S) at the University of Texas Southwestern Medical Center (UTSW). All work described was performed according to the current guidelines for animal care and handling biohazard agents.

### Sepsis Protocol

Adult Sprague Dawley (SD) male rats (325–360 g, Harlan Laboratories, Houston, TX) were allowed 5–10 days to acclimate to surroundings after arrival. Sepsis was induced by intratracheal injection of *Streptococcus pneumoniae* type 3 (ATCC 6303, Rockville, MD), 4×10^6^ colony forming units (CFU) per rat.

To amplify bacteria, *S. pneumoniae* were reconstituted and injected into the lungs of a rat to increase their virulence; lung lavage liquid was plated and purified, and aliquots were prepared and stored at −80°C until use. Before each experiment, individual aliquots were thawed and amplified on trypticase soy blood agar plates overnight at 37°C. Bacteria were collected with sterile endotoxin-free phosphate-buffered saline (PBS). The broth was centrifuged and the resultant pellet was washed twice with PBS to remove any impurities adherent to the bacteria. The bacteria were then re-suspended in PBS, agitated, and drawn up into sterile tuberculin syringes in aliquots. Bacterial CFU numbers were determined by plating 100 µl of the bacterial suspension onto blood agar plates in serial dilutions and incubating the plates overnight at 37°C.

To induce aspiration pneumonia, rats were anesthetized with isoflurane and placed in a supine position. The area over the trachea was prepared with 10% povidine-iodine solution. A midline cervical incision was made, and the trachea was identified and isolated via blunt dissection. A 0.4-ml aliquot of either sterile endotoxin-free PBS or bacterial suspension (4×10^6^ CFU) was injected directly into the trachea. After the wound was closed with surgical staples, the animals were placed on a 30° incline to ensure accumulation of the injected fluid into the lungs. All rats were given 10 ml of lactated Ringer’s solution intraperitoneally while anesthetized to ensure hydration. Our previous studies have shown that the surgical procedure alone (injection of PBS and no bacteria) produces no ill effects.

### Preparation of Tissue Lysates and Cellular Fractions

Animals were sacrificed and heart tissues were harvested, washed in PBS, snap clamp frozen, and kept at −80°C. Tissue lysates were prepared using tissue protein extraction reagent (Thermo Fisher Scientific, Rockford, IL), and mitochondrial and cytosolic fractions were separated by differential centrifugation using the mitochondria isolation kit for tissue (Sigma-Aldrich, Saint Louis, MO) according to manufacturer protocols. All collected samples were stored as aliquots at −80°C until used.

### Proteinase K Digestion of Mitochondrial Fractions

According to published methods [21], [67], mitochondrial preparations were treated with proteinase K, 100 ng/ml, in mitochondrial isolation buffer without EDTA in the absence or presence of 0.5% Triton X-100. After incubation at room temperature for 30 min, the reaction was stopped by the addition of protease inhibitor cocktail (all reagents from Sigma-Aldrich, St. Louis, MO).

### Western Blot

SDS-PAGE gel samples were prepared using 2X sample buffer (Sigma-Aldrich, St. Louis, MO) and loaded to 4–15% gradient gels at 20 µg/lane. Proteins were transferred to PVDF membranes (BioRad, Hercules, CA), blocked with 5% nonfat milk-PBS at room temperature for 1 hour and subsequently probed with primary antibodies. The membranes were then rinsed and incubated with corresponding horseradish peroxidase conjugated secondary antibodies. Antibody dilutions and incubation time were chosen according to manufacturer’s instructions. Signals were detected by using SuperSignal West Pico chemiluminescent substrate (Thermo Scientific, Asheville, NC). Antibodies used in this report were purchased from the following vendors: antibody cocktails against rodent mitochondrial membrane integrity and total OXPHOX antibody were from Abcam, Cambridge, MA, anti-GAPDH was from Millipore, Billerica, MA, and the other antibodies were from Santa Cruz Biotechnology, Santa Cruz, CA. Secondary antibodies were from Bio-Rad, Hercules, CA.

### Real-time PCR (RPCR)

Total RNA from the heart tissue was isolated using RNeasy Mini kit (Qiagen, Valencia, CA), quantified by a NanoDrop spectrophotometer (NanoDrop Inc., Wilmington, DE), and cDNA was subsequently synthesized using a High Capacity cDNA Reverse Transcription Kit (Life Technologies Corporation, Carlsbad, CA) according to manufacturers suggested protocols. Primers and TaqMan probes (FAM dye-labeled) for Src, SHP2 or GAPDH were added to the cDNA to start RPCR amplification, and products were measured using an ABI HT7900 Real-time PCR System (Life Technologies Corporation, Carlsbad, CA). The threshold cycle (Ct) was obtained from triplicate samples and averaged. The ΔCt was calculated as the difference between the Cts for a target gene and housekeeping gene GAPDH, whereas the ΔΔCt was the difference between the ΔCts for sham and sepsis samples. The data were expressed as a relative quantification, in which sepsis-induced fold change of a target gene expression was calculated as 2^−ΔΔCt^.

### Immunoprecipitation

Mitochondrial pellets were re-suspended in PBS and solubilized with 1 mM n-dodecyl-ß-D-maltopyranoside (Abcam, Cambridge, MA) on ice for 30 min. Each 0.5-mg protein sample was incubated with 50 µl anti-phosphotyrosine-sepharose 4B (Invitrogen, Carlsbad, CA) or 25 µl mitochondrial complex (I-V) immunocapture antibody (Abcam, Cambridge, MA) at 4°C overnight. The precipitates were washed 3 times with cold PBS and proteins were solubilized with 30 µl 2X SDS sample buffer. The resulted samples were loaded on SDS-PAGE gels for Western blot analysis.

### *In vitro* Reaction of Recombinant Active Src and SHP2 with Mitochondria Proteins

Mitochondrial pellets were re-suspended in PBS and solubilized with 1 mM n-dodecyl-ß-D-maltopyranoside on ice for 30 min. Recombinant human active Src or SHP2 (R&D Systems, Inc., Minneapolis, MN) were set to treat mitochondria preparations according to the conditions recommended by manufacturer protocols. In a reaction that required active Src, 2 mg/ml mitochondria fraction and 40 µg/ml Src were added to 25 µl kinase buffer (5 mM MOPS, pH 7.2, 2.5 mM ß-glycerolphosphate, 4 mM MgCl_2_, 2.5 mM MnCl_2_, 1 mM EGTA, 0.4 mM EDTA, and 50 nM DTT) followed by a 15-minute incubation at 30°C. In the case that active SHP2 was used, 1 mg/ml mitochondria fraction and 80 µg/ml SHP2 were added to 50 µl phosphatase buffer (8 mM HEPES, 80 nM EDTA, 80 mM EGTA, 4 mg/ml BSA and 80 nM DTT) followed by a 30-minute incubation at 37°C.

### Mitochondrial OXPHOS Activities

Complex I, II-III, IV or V activities were measured using enzyme assay kits (Abcam, Cambridge, MA) according to manufacturer’s protocols. 50–100 µg mitochondrial fractions were used in each reaction. Individual enzymatic activity was determined as change of absorbance per minute per µg mitochondrial protein at wavelength 450 nm for complex I, 550 nm for complex II-III and complex IV, and 340 nm for complex V. All measurements were performed in at least duplicate.

### Measurements of Mitochondrial ROS Production

Mitochondrial H_2_O_2_ release was determined according to a previously published protocol [45], [50]. Briefly, 40 µg fresh mitochondrial preparations were set to react with 5 µM Amplex Red and 0.1 U/ml horseradish peroxidase (HRP) (Invitrogen, Grand Island, NY) in 200 µl reaction buffer (in mM: 125 KCl, 10 MOPS, 2 MgSO_4_, 2 KH_2_PO_4_, 10 NaCl, 1 EGTA, 0.7 CaCl_2_, pH7.2). Superoxide dismutase (SOD) (Sigma-Aldrich, Saint Louis, MO) was added at 50 U/ml to convert all superoxide into H_2_O_2_. Mitochondrial respiration substrate succinate (5 mM) was added to start the reaction. The reaction mixture was then incubated in the dark at 37°C for 30 minutes. HRP catalyzed H_2_O_2_-dependent oxidation of Amplex Red, the end product Resorufin Red, was measured by fluorescent reading at Ex/Em = 570/620 (PHERAstar, BMG LABTECH, Cary, NC). All measurements were performed in at least duplicate. H_2_O_2_ concentrations were calculated according to a standard curve.

### Mitochondrial Aconitase Assay

The colometric aconitase assay (BioVision, Mountain View, CA) was used to determine aconitase activities in the mitochondrial fractions. In each reaction, 100 µg mitochondrial protein was applied and aconitase activity was determined via converting citrate to isocitrate according to the manufacturer’s protocol. The final product was measured at wavelength 450 nm, and all measurements were performed in at least duplicate.

### Statistical Analysis

All data were expressed as mean ± SEM of at least 3 independent experiments using 4–8 animals/group. Student’s t-tests were used to assess the difference between the sham and sepsis groups and the groups with or without a treatment. Probability values less than 0.05 were considered statistically significant (analyses were performed using SPSS for Windows, Version 7.5.1).

## References

[1] Angus DC, Pereira CA, Silva E (2006) Epidemiology of severe sepsis around the world. Endocr Metab Immune Disord Drug Targets 6: 207–212. 16787296 10.2174/187153006777442332

[2] Bone RC, Balk RA, Cerra FB, Dellinger RP, Fein AM, et al (1992) Definitions for sepsis and organ failure and guidelines for the use of innovative therapies in sepsis. The ACCP/SCCM Consensus Conference Committee. American College of Chest Physicians/Society of Critical Care Medicine. Chest 101: 1644–1655. 1303622 10.1378/chest.101.6.1644

[3] Levy MM, Dellinger RP, Townsend SR, Linde-Zwirble WT, Marshall JC, et al. (20120) The Surviving Sepsis Campaign: results of an international guideline-based performance improvement program targeting severe sepsis. Crit Care Med 38: 367–374. 10.1097/CCM.0b013e3181cb0cdc 20035219

[4] O’Brien JM Jr, Ali NA, Aberegg SK, Abraham E (2007) Sepsis. Am J Med 120: 1012–1022. 18060918 10.1016/j.amjmed.2007.01.035

[5] Crouser ED (2004) Mitochondrial dysfunction in septic shock and multiple organ dysfunction syndrome. Mitochondrion 4: 729–741. 16120428 10.1016/j.mito.2004.07.023

[6] Ruggieri AJ, Levy RJ, Deutschman CS (2010) Mitochondrial dysfunction and resuscitation in sepsis. Crit Care Clin 26: 567–575, x–xi. 10.1016/j.ccc.2010.04.007 PMC2908601 20643307

[7] Brealey D, Brand M, Hargreaves I, Heales S, Land J, et al (2002) Association between mitochondrial dysfunction and severity and outcome of septic shock. Lancet 360: 219–223. 12133657 10.1016/S0140-6736(02)09459-X

[8] Cairns CB, Moore FA, Haenel JB, Gallea BL, Ortner JP, et al (1997) Evidence for early supply independent mitochondrial dysfunction in patients developing multiple organ failure after trauma. J Trauma 42: 532–536. 9095123 10.1097/00005373-199703000-00023

[9] Fink MP (2002) Bench-to-bedside review: Cytopathic hypoxia. Crit Care 6: 491–499. 12493070 10.1186/cc1824 PMC153437

[10] Fink MP (2001) Cytopathic hypoxia. Mitochondrial dysfunction as mechanism contributing to organ dysfunction in sepsis. Crit Care Clin 17: 219–237. 11219231 10.1016/s0749-0704(05)70161-5

[11] Tsutsui H, Kinugawa S, Matsushima S (2008) Oxidative stress and mitochondrial DNA damage in heart failure. Circ J 72 Suppl A: A31–37. 10.1253/circj.cj-08-0014 18772530

[12] Ballinger SW (2005) Mitochondrial dysfunction in cardiovascular disease. Free Radic Biol Med 38: 1278–1295. 15855047 10.1016/j.freeradbiomed.2005.02.014

[13] Han D, Williams E, Cadenas E (2001) Mitochondrial respiratory chain-dependent generation of superoxide anion and its release into the intermembrane space. Biochem J 353: 411–416. 11139407 10.1042/0264-6021:3530411 PMC1221585

[14] Wang Y, Zang QS, Liu Z, Wu Q, Maass D, et al (2011) Regulation of VEGF-induced endothelial cell migration by mitochondrial reactive oxygen species. Am J Physiol Cell Physiol 301: C695–704. 21653897 10.1152/ajpcell.00322.2010 PMC3174570

[15] Dikalova AE, Bikineyeva AT, Budzyn K, Nazarewicz RR, McCann L, et al (2010) Therapeutic targeting of mitochondrial superoxide in hypertension. Circ Res 107: 106–116. 20448215 10.1161/CIRCRESAHA.109.214601 PMC2901409

[16] Zhou R, Yazdi AS, Menu P, Tschopp J (2011) A role for mitochondria in NLRP3 inflammasome activation. Nature 469: 221–225. 21124315 10.1038/nature09663

[17] Seth RB, Sun L, Ea CK, Chen ZJ (2005) Identification and characterization of MAVS, a mitochondrial antiviral signaling protein that activates NF-kappaB and IRF 3. Cell 122: 669–682. 16125763 10.1016/j.cell.2005.08.012

[18] Zhang Q, Raoof M, Chen Y, Sumi Y, Sursal T, et al (2010) Circulating mitochondrial DAMPs cause inflammatory responses to injury. Nature 464: 104–107. 20203610 10.1038/nature08780 PMC2843437

[19] Feng J, Lucchinetti E, Enkavi G, Wang Y, Gehrig P, et al (2010) Tyrosine phosphorylation by Src within the cavity of the adenine nucleotide translocase 1 regulates ADP/ATP exchange in mitochondria. Am J Physiol Cell Physiol 298: C740–748. 20007455 10.1152/ajpcell.00310.2009 PMC2838572

[20] Arachiche A, Augereau O, Decossas M, Pertuiset C, Gontier E, et al (2008) Localization of PTP-1B, SHP-2, and Src exclusively in rat brain mitochondria and functional consequences. J Biol Chem 283: 24406–24411. 18583343 10.1074/jbc.M709217200 PMC3259839

[21] Lewandrowski U, Sickmann A, Cesaro L, Brunati AM, Toninello A, et al (2008) Identification of new tyrosine phosphorylated proteins in rat brain mitochondria. FEBS Lett 582: 1104–1110. 18331841 10.1016/j.febslet.2008.02.077

[22] Monick MM, Powers LS, Barrett CW, Hinde S, Ashare A, et al (2008) Constitutive ERK MAPK activity regulates macrophage ATP production and mitochondrial integrity. J Immunol 180: 7485–7496. 18490749 10.4049/jimmunol.180.11.7485 PMC2410094

[23] Augereau O, Claverol S, Boudes N, Basurko MJ, Bonneu M, et al (2005) Identification of tyrosine-phosphorylated proteins of the mitochondrial oxidative phosphorylation machinery. Cell Mol Life Sci 62: 1478–1488. 15924266 10.1007/s00018-005-5005-7 PMC11139224

[24] Deng N, Zhang J, Zong C, Wang Y, Lu H, et al.. (2011) Phosphoproteome analysis reveals regulatory sites in major pathways of cardiac mitochondria. Mol Cell Proteomics 10: M110 000117. 10.1074/mcp.M110.000117 PMC3033665 20495213

[25] Tibaldi E, Brunati AM, Massimino ML, Stringaro A, Colone M, et al (2008) Src-Tyrosine kinases are major agents in mitochondrial tyrosine phosphorylation. J Cell Biochem 104: 840–849. 18247338 10.1002/jcb.21670

[26] Budas GR, Churchill EN, Disatnik MH, Sun L, Mochly-Rosen D (2010) Mitochondrial import of PKCepsilon is mediated by HSP90: a role in cardioprotection from ischaemia and reperfusion injury. Cardiovasc Res 88: 83–92. 20558438 10.1093/cvr/cvq154 PMC2936125

[27] Guo J, Cong L, Rybin VO, Gertsberg Z, Steinberg SF (2010) Protein kinase C-{delta} regulates the subcellular localization of Shc in H2O2-treated cardiomyocytes. Am J Physiol Cell Physiol 299: C770–778. 20686066 10.1152/ajpcell.00170.2010 PMC2957271

[28] Alonso M, Melani M, Converso D, Jaitovich A, Paz C, et al (2004) Mitochondrial extracellular signal-regulated kinases 1/2 (ERK1/2) are modulated during brain development. J Neurochem 89: 248–256. 15030409 10.1111/j.1471-4159.2004.02323.x

[29] Miyazaki T, Neff L, Tanaka S, Horne WC, Baron R (2003) Regulation of cytochrome c oxidase activity by c-Src in osteoclasts. J Cell Biol 160: 709–718. 12615910 10.1083/jcb.200209098 PMC2173369

[30] Court O, Kumar A, Parrillo JE (2002) Clinical review: Myocardial depression in sepsis and septic shock. Crit Care 6: 500–508. 12493071 10.1186/cc1822 PMC153435

[31] Zanotti-Cavazzoni SL, Hollenberg SM (2009) Cardiac dysfunction in severe sepsis and septic shock. Curr Opin Crit Care 15: 392–397. 19633546 10.1097/MCC.0b013e3283307a4e

[32] Rudiger A, Singer M (2007) Mechanisms of sepsis-induced cardiac dysfunction. Crit Care Med 35: 1599–1608. 17452940 10.1097/01.CCM.0000266683.64081.02

[33] Blanco J, Muriel-Bombin A, Sagredo V, Taboada F, Gandia F, et al (2008) Incidence, organ dysfunction and mortality in severe sepsis: a Spanish multicentre study. Crit Care 12: R158. 19091069 10.1186/cc7157 PMC2646323

[34] Parrillo JE, Parker MM, Natanson C, Suffredini AF, Danner RL, et al (1990) Septic shock in humans. Advances in the understanding of pathogenesis, cardiovascular dysfunction, and therapy. Ann Intern Med 113: 227–242. 2197912 10.7326/0003-4819-113-3-227

[35] Kayar SR, Banchero N (1987) Volume density and distribution of mitochondria in myocardial growth and hypertrophy. Respir Physiol 70: 275–286. 2961036 10.1016/0034-5687(87)90010-7

[36] Watts JA, Kline JA, Thornton LR, Grattan RM, Brar SS (2004) Metabolic dysfunction and depletion of mitochondria in hearts of septic rats. J Mol Cell Cardiol 36: 141–150. 14734056 10.1016/j.yjmcc.2003.10.015

[37] Levy RJ (2007) Mitochondrial dysfunction, bioenergetic impairment, and metabolic down-regulation in sepsis. Shock 28: 24–28. 17483747 10.1097/01.shk.0000235089.30550.2d

[38] Callahan LA, Supinski GS (2007) Diaphragm and cardiac mitochondrial creatine kinases are impaired in sepsis. J Appl Physiol 102: 44–53. 16916915 10.1152/japplphysiol.01204.2005

[39] Greenhalgh DG, Saffle JR, Holmes JHt, Gamelli RL, Palmieri TL, et al (2007) American Burn Association consensus conference to define sepsis and infection in burns. J Burn Care Res 28: 776–790. 17925660 10.1097/BCR.0b013e3181599bc9

[40] Wang L, Quan J, Johnston WE, Maass DL, Horton JW, et al (2010) Age-dependent differences of interleukin-6 activity in cardiac function after burn complicated by sepsis. Burns 36: 232–238. 19501973 10.1016/j.burns.2009.02.019 PMC2823965

[41] Sheeran PW, Maass DL, White DJ, Turbeville TD, Giroir BP, et al (1998) Aspiration pneumonia-induced sepsis increases cardiac dysfunction after burn trauma. J Surg Res 76: 192–199. 9698522 10.1006/jsre.1998.5352

[42] White J, Thomas J, Maass DL, Horton JW (2003) Cardiac effects of burn injury complicated by aspiration pneumonia-induced sepsis. Am J Physiol Heart Circ Physiol 285: H47–58. 12637356 10.1152/ajpheart.00833.2002

[43] Tao W, Maass DL, Johnston WE, Horton JW (2005) Murine in vivo myocardial contractile dysfunction after burn injury is exacerbated by pneumonia sepsis. Shock 24: 495–499. 16247338 10.1097/01.shk.0000183431.78973.cd

[44] Zang Q, Maass DL, Tsai SJ, Horton JW (2007) Cardiac mitochondrial damage and inflammation responses in sepsis. Surg Infect (Larchmt) 8: 41–54. 17381396 10.1089/sur.2006.033 PMC6044285

[45] Zang QS, Sadek H, Maass DL, Martinez B, Ma L, et al (2012) Specific inhibition of mitochondrial oxidative stress suppresses inflammation and improves cardiac function in a rat pneumonia-related sepsis model. Am J Physiol Heart Circ Physiol 302: H1847–1859. 22408027 10.1152/ajpheart.00203.2011

[46] Vyssokikh MY, Brdiczka D (2003) The function of complexes between the outer mitochondrial membrane pore (VDAC) and the adenine nucleotide translocase in regulation of energy metabolism and apoptosis. Acta Biochim Pol 50: 389–404. 12833165

[47] Lunardi J, Attardi G (1991) Differential regulation of expression of the multiple ADP/ATP translocase genes in human cells. J Biol Chem 266: 16534–16540. 1885585

[48] Zang QS, Maass DL, Wigginton JG, Barber RC, Martinez B, et al (2010) Burn serum causes a CD14-dependent mitochondrial damage in primary cardiomyocytes. Am J Physiol Heart Circ Physiol 298: H1951–1958. 20348223 10.1152/ajpheart.00927.2009 PMC2886640

[49] Zang Q, Maass DL, White J, Horton JW (2007) Cardiac mitochondrial damage and loss of ROS defense after burn injury: the beneficial effects of antioxidant therapy. J Appl Physiol 102: 103–112. 16931562 10.1152/japplphysiol.00359.2006 PMC6044277

[50] Starkov AA (2010) Measurement of mitochondrial ROS production. Methods Mol Biol 648: 245–255. 20700717 10.1007/978-1-60761-756-3_16 PMC3057530

[51] Korge P, Ping P, Weiss JN (2008) Reactive oxygen species production in energized cardiac mitochondria during hypoxia/reoxygenation: modulation by nitric oxide. Circ Res 103: 873–880. 18776040 10.1161/CIRCRESAHA.108.180869 PMC2755534

[52] Deng N, Zhang J, Zong C, Wang Y, Lu H, et al.. (2011) Phosphoproteome analysis reveals regulatory sites in major pathways of cardiac mitochondria. Mol Cell Proteomics 10: M110 000117. 10.1074/mcp.M110.000117 PMC3033665 20495213

[53] Di Pancrazio F, Bisetto E, Alverdi V, Mavelli I, Esposito G, et al (2006) Differential steady-state tyrosine phosphorylation of two oligomeric forms of mitochondrial F0F1ATPsynthase: a structural proteomic analysis. Proteomics 6: 921–926. 16400683 10.1002/pmic.200500077

[54] Fullerton JN, Singer M (2011) Organ failure in the ICU: cellular alterations. Semin Respir Crit Care Med 32: 581–586. 21989694 10.1055/s-0031-1287866

[55] Japiassu AM, Santiago AP, d’Avila JC, Garcia-Souza LF, Galina A, et al (2011) Bioenergetic failure of human peripheral blood monocytes in patients with septic shock is mediated by reduced F1Fo adenosine-5′-triphosphate synthase activity. Crit Care Med 39: 1056–1063. 21336129 10.1097/CCM.0b013e31820eda5c

[56] Jakob S, Altschmied J, Haendeler J (2009) “Shping 2” different cellular localizations - a potential new player in aging processes. Aging (Albany NY) 1: 664–668. 20157547 10.18632/aging.100063 PMC2806037

[57] Hebert-Chatelain E, Jose C, Gutierrez Cortes N, Dupuy JW, Rocher C, et al (2012) Preservation of NADH ubiquinone-oxidoreductase activity by Src kinase-mediated phosphorylation of NDUFB10. Biochim Biophys Acta 1817: 718–725. 22321370 10.1016/j.bbabio.2012.01.014

[58] Hebert Chatelain E, Dupuy JW, Letellier T, Dachary-Prigent J (2011) Functional impact of PTP1B-mediated Src regulation on oxidative phosphorylation in rat brain mitochondria. Cell Mol Life Sci 68: 2603–2613. 21063895 10.1007/s00018-010-0573-6 PMC11115002

[59] Chen Q, Vazquez EJ, Moghaddas S, Hoppel CL, Lesnefsky EJ (2003) Production of reactive oxygen species by mitochondria: central role of complex III. J Biol Chem 278: 36027–36031. 12840017 10.1074/jbc.M304854200

[60] Salvi M, Morrice NA, Brunati AM, Toninello A (2007) Identification of the flavoprotein of succinate dehydrogenase and aconitase as in vitro mitochondrial substrates of Fgr tyrosine kinase. FEBS Lett 581: 5579–5585. 17997986 10.1016/j.febslet.2007.11.005

[61] Chou HC, Chen YW, Lee TR, Wu FS, Chan HT, et al (2010) Proteomics study of oxidative stress and Src kinase inhibition in H9C2 cardiomyocytes: a cell model of heart ischemia-reperfusion injury and treatment. Free Radic Biol Med 49: 96–108. 20385227 10.1016/j.freeradbiomed.2010.04.001

[62] Puceat M, Roche S, Vassort G (1998) Src family tyrosine kinase regulates intracellular pH in cardiomyocytes. J Cell Biol 141: 1637–1646. 9647655 10.1083/jcb.141.7.1637 PMC2133004

[63] Melendez J, Turner C, Avraham H, Steinberg SF, Schaefer E, et al (2004) Cardiomyocyte apoptosis triggered by RAFTK/pyk2 via Src kinase is antagonized by paxillin. J Biol Chem 279: 53516–53523. 15322113 10.1074/jbc.M408475200

[64] Gu Y, Zou Y, Aikawa R, Hayashi D, Kudoh S, et al (2001) Growth hormone signalling and apoptosis in neonatal rat cardiomyocytes. Mol Cell Biochem 223: 35–46. 11681720 10.1023/a:1017941625858

[65] Sabri A, Guo J, Elouardighi H, Darrow AL, Andrade-Gordon P, et al (2003) Mechanisms of protease-activated receptor-4 actions in cardiomyocytes. Role of Src tyrosine kinase. J Biol Chem 278: 11714–11720. 12522105 10.1074/jbc.M213091200

[66] Zou Y, Komuro I, Yamazaki T, Kudoh S, Uozumi H, et al (1999) Both Gs and Gi proteins are critically involved in isoproterenol-induced cardiomyocyte hypertrophy. J Biol Chem 274: 9760–9770. 10092665 10.1074/jbc.274.14.9760

[67] Salvi M, Brunati AM, Bordin L, La Rocca N, Clari G, et al (2002) Characterization and location of Src-dependent tyrosine phosphorylation in rat brain mitochondria. Biochim Biophys Acta 1589: 181–195. 12007793 10.1016/s0167-4889(02)00174-x

